# PHYSICAL ACTIVITY AT THE INTERSECTIONS OF AGING AND DISABILITY

**DOI:** 10.1093/geroni/igac059.061

**Published:** 2022-12-20

**Authors:** Annalisa Na, Annalisa Na

## Abstract

Physical activity is beneficial for older adults to maintain health and help manage chronic diseases but relies on routine participation. Some older adults continue with physical activity behaviors as they age, whereas others are negatively affected by impaired functional abilities, physical disabilities, and unmet psychosocial needs. This symposium presents quantitative and qualitative data on physical and psychosocial factors that can influence physical activity among older adults with varying levels and types of physical abilities. The first presentation will focus on aging into the disability associated with knee osteoarthritis, the leading cause of mobility decline among older adults that generally onsets after the fifth decade of life. The second presentation will focus on aging with a disability, specifically how health perspectives evolve when aging with a spinal cord injury. The third presentation will highlight how the COVID-19 pandemic has influenced outdoor physical activity among older adults when indoor activities became limited. The fourth presentation shifts perspectives to clinicians, specifically physical therapists, to explore the needs for addressing physical activity in the clinic. The fifth presentation outlines a physical activity intervention program and its implementation for community-dwelling older adults. Together, these presentations will provide practical insights for designing a person-centered program to improve physical activity for older adults including those aging into disability or aging with disability.

